# Biotechnologically Engineered Plants

**DOI:** 10.3390/biology12040601

**Published:** 2023-04-15

**Authors:** Zareen Narayanan, Bernard R. Glick

**Affiliations:** 1 Division of Biological Sciences, School of STEM, University of Washington, Bothell, WA 98011, USA; 2 Department of Biology, University of Waterloo, Waterloo, ON N2L3G1, Canada; glick@uwaterloo.ca

**Keywords:** transgenic plants, commercialized plants, recombinant proteins, plants producing pharmaceuticals, nutrients, fruit color, disease resistance, vaccines

## Abstract

**Simple Summary:**

Scientists are exploring the use of new genetic tools to engineer crop varieties which can result in desirable traits such as improved yield, disease resistance, and enhanced nutritional value. By manipulating the plants’ genetic makeup, they are able to develop transgenic plants capable of producing valuable biotechnological compounds. The development of plant-based biomanufacturing could offer a more sustainable and cost-effective method to produce useful products compared to traditional manufacturing process.

**Abstract:**

The development of recombinant DNA technology during the past thirty years has enabled scientists to isolate, characterize, and manipulate a myriad of different animal, bacterial, and plant genes. This has, in turn, led to the commercialization of hundreds of useful products that have significantly improved human health and well-being. Commercially, these products have been mostly produced in bacterial, fungal, or animal cells grown in culture. More recently, scientists have begun to develop a wide range of transgenic plants that produce numerous useful compounds. The perceived advantage of producing foreign compounds in plants is that compared to other methods of producing these compounds, plants seemingly provide a much less expensive means of production. A few plant-produced compounds are already commercially available; however, many more are in the production pipeline.

## 1. Introduction

**Figure 1:**
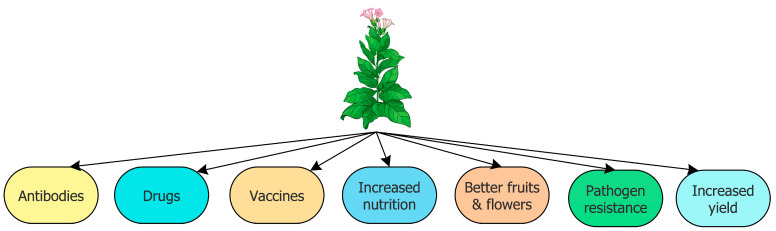
Schematic representation of some transgenic plants and the products that they produce.

Plant biotechnology has two main objectives: (i) the use of plants as a means of producing a wide range of products that have been previously synthesized using recombinant bacteria, fungi, or animal cells in culture, and (ii) the creation of new, unique, and better-performing varieties of crop plants. Some of the new and unique varieties of crop plants include plants resistant to insects, viruses, and herbicides; plants with increased nutritional content; plants that are resistant to a range of abiotic environmental stresses such as high levels of soil salinity and drought; plants with modified fruits and flowers; plants that are resistant to a wide range of fungal and bacterial pathogens; and plants with a significant increase in yield. In addition, the most prominent products that are either currently produced in plants or are being developed for eventual production in plants include antibodies and antibody fragments; human and animal vaccines; and a wide range of animal and human therapeutic agents (Figure 1).

## 2. Early Engineered Plants

Plants that are resistant to insect predation, damage from plant viruses, and various herbicides (used to prevent the growth of weeds) were genetically engineered beginning ~30–40 years ago, and a number of these engineered plants have been commercially available for many years. Thus, these plants are only mentioned briefly in this review, where the focus is on the development of some of the more recent genetically modified plants.

### 2.1. Insect Resistance

Beginning with the 1940s through the 1970s, several powerful chemical insecticides were developed and employed globally on a massive scale. The use of these insecticides had a dramatic effect in reducing the damage to crop plants that resulted from insect predation. The most effective and most widely use of these chemical insecticides was the compound dichlorodiphenyltrichloroethane or DDT [1]. Unfortunately, it was eventually discovered that most of these chemical insecticides, and DDT in particular, had negative effects on animals, ecosystems, and humans [2]. Moreover, many of these chemicals, notwithstanding their apparent effectiveness as insecticides, persisted for many years in the environment and accumulated in increasing concentrations through food chains. With the widespread development of transgenic plants in the early 1980s, scientists began developing plants that were resistant to insect predation [3,4,5,6,7,8,9,10]. For the most part, this involved isolating genes encoding bacterial insect-toxigenic proteins from various strains of the bacterium *Bacillus thuringiensis* and expressing those genes in transgenic plants; this approach has turned out to be highly effective and without significant negative consequences. In the past 25 years, the availability and commercial use of transgenic plants expressing one or more of several different *B. thuringiensis* insecticidal toxins has increased dramatically throughout the US and many other countries of the world [11]. At the present time, 44 countries worldwide (with the notable exception of countries belonging to the European Union) have given regulatory approval to 40 different genetically engineered crops with insect and herbicide-resistant plants being the most commonly introduced traits [12]. Currently, regulatory approved *B. thuringiensis*-engineered plants include cotton, cowpea, eggplant, maize, poplar, potato, rice, soybean, sugarcane, and tomato.

### 2.2. Virus Resistance

It has been estimated that there are nearly 2000 known different plant viruses [13], the great majority of which have a small single-stranded RNA genome (often less than 10 kilobases). These viruses can cause significant damage to crop plants, thereby dramatically reducing plant yields. The viral coat protein is generally the most abundant protein in small single-stranded RNA viruses. Consequently, many transgenic plants have been engineered to express a small single-stranded RNA viral coat protein gene and, as a result are often protected against the systematic spread and the subsequent deleterious effects of that virus [14,15,16,17,18,19,20]. It is believed that the cloned and expressed viral coat protein gene inhibits the expression of the small single-stranded RNA virus through the mechanism of RNA interference (RNAi). In addition, given the similarity in structure and mechanisms of infectivity of many small plant viruses, the abovementioned viral coat protein gene approach has been found to be highly effective for many different plants, including alfalfa, corn, tobacco, tomato, sugar beet, cucumber, papaya, potato, rice, zucchini, soybean, grapevine, squash, pumpkin, plum, and muskmelon, against several different viruses. Moreover, numerous transgenic plants expressing a small single-stranded RNA viral coat protein gene have been approved by regulatory authorities and subsequently commercialized. Unfortunately, the viral coat protein strategy is only effective when transgenic plants are challenged by closely related viruses. Therefore, scientists have sought to develop transgenic plants that are resistant to a broad spectrum of plant viruses. While several different approaches have been attempted to achieve this end, this remains a work in progress.

### 2.3. Herbicide Resistance

Every year a large portion of worldwide crop production is lost through weed infestation. This loss of productivity notwithstanding, an estimated ~USD 35–40 billion is spent annually by farmers on >100 different chemical herbicides [21]. Since most herbicides do not discriminate between weeds and crop plants, scientists have developed several different herbicide-resistant crop plants, including plants resistant to triazines, sulfonylureas, imidazolinones, aryloxphenoxypropionates, cyclohexanediones, bromoxynil, phenoxycarboxylic acids, glufosinate, cyanamide, and dalapon. This approach has been so successful that currently ~60% of the transgenic crops that are planted globally have been modified to be herbicide-resistant. However, herbicide-resistant crops do not have a higher yield than non-herbicide-resistant crops [22]. On the other hand, herbicide-resistant crops are preferred for “improved and simplified weed control, less labour and fuel cost, no-till planting/planting flexibility, yield increase, extended time window for spraying, and in some cases decreased pesticide input” [23].

Worldwide, the most widely used chemical herbicide is glyphosate (commercially sold as Roundup^®^(Bayer, USA), which is claimed by its producer (the Monsanto Corporation, now a division of Bayer) to be safe, cheap, effective, and environmentally friendly [24]. Transgenic plants that have been shown to be resistant to this herbicide include soybean, corn, canola, tobacco, soybean, petunia, tomato, potato, alfalfa, sorghum, sugar beet, Indian mustard, and cotton. Treatment of plants in the field with glyphosate should kill all (or most) of the weeds while the engineered crop plants are not affected. With the widespread acceptance of this herbicide there is, however, some concern that (i) there is too much dependence upon the use of a single herbicide and, as a result, there are numerous instances of weeds acquiring naturally occurring resistance [25,26]; and (ii) some reports have suggested that long term exposure to this herbicide may be toxic or carcinogenic to humans [27]. Thus, there are some recent literature reports of plants that have been engineered to be resistant to other herbicides that researchers hope will provide an effective alternative to the widespread use of glyphosate [28,29,30]. However, glyphosate is likely to continue to be the dominant herbicide worldwide until some of these newer approaches can be shown to safe and efficacious in the field with a large number of different transgenic plants [31].

## 3. Antibody Production

Currently, therapeutic monoclonal antibodies are mostly produced from transgenic Chinese hamster ovary (CHO) cells grown in culture, typically in stirred tank bioreactors with volumes as large as 25,000 L. Unfortunately, this process, which requires highly specialized fermentation equipment and media, and highly controlled conditions, yields only a modest level of monoclonal antibody protein per run (which takes around two weeks’ time). Producing monoclonal antibodies from transgenic CHO cells grown in culture results in an average monoclonal antibody treatment retailing for nearly USD 100,000 per annum [32] with production cost being estimated to be from USD 100 to 200 per gram. This results in the average treatment with a monoclonal antibody costing between USD 15,000 and USD 200,000 per year. On the other hand, with current technology, functional monoclonal antibodies can be produced in transgenic plants at only 1–10% of the cost of using CHO cells, notwithstanding the fact that it takes longer to grow transgenic plants than it does to produce monoclonal antibodies from CHO cells in culture. As an alternative to transgenic plants, transient gene expression can produce a high protein yield in a much shorter period of time [33]. In addition, unlike the requirement to grow CHO cells in very expensive large fermenters, plants can easily be grown on a relatively large scale in semi-contained and less expensive greenhouse facilities.

The majority of human proteins, including human antibodies, are glycosylated, with sugar molecules covalently attached to either a hydroxyl group of an antibody molecule serine or threonine residue, or to an amide moiety of an asparagine residue [34,35,36,37]. There are significant differences in the patterns of protein glycosylation produced by humans versus plants. That is, humans and plants add different carbohydrate molecules to the surface-facing amino acids of antibody molecules. While differences in surface carbohydrate molecules do not alter the activity of the antibody molecules, “other properties, such as protein folding, stability, solubility, susceptibility to proteases, blood clearance rate, and antigenicity, can be affected profoundly” [38]. To overcome any potential problems that might occur as a consequence of using incorrectly glycosylated plant-produced monoclonal antibodies, scientists have preemptively genetically engineered several plant hosts to glycosylate foreign proteins produced in those plants so that the glycosylation pattern of plant-produced proteins is nearly identical to the glycosylation pattern of the same proteins produced in human cells [36,39]. According to Castilho and Steinkellner [36], “Plants have demonstrated a high degree of tolerance to changes in the *N*-glycosylation pathway, allowing recombinant proteins to be modified into human-like structures in a specific and controlled manner.” Thus, in the correct genetic background, plant-produced antibodies do not show any significant differences in their ability to bind to their target antigens.

In early experiments, scientists synthesized cDNAs (separately encoding light and heavy antibody chains) that were previously isolated from mouse hybridoma mRNAs and expressed these cDNAs in transgenic tobacco plants [40]. Plants that expressed single (light or heavy) chains from the same antibody molecule were then genetically crossed with one another to produce progeny in which both light and heavy chains were produced in the same plant, with the hope that these chains would correctly associate with one another to form functional complete antibody molecules. Notwithstanding this long and somewhat cumbersome process, some transgenic tobacco plants producing functional antibodies were obtained. Since that time, several more efficient systems have been developed for the synthesis of monoclonal antibodies in plants.

Table 1 summarizes some of the full-size monoclonal antibodies that have been produced in plants [39,41,42,43,44,45,46,47,48]. In addition, this table does not include the numerous plant-synthesized antibody fragments or single chain antibodies that have been produced. Since scientists can produce full-size active monoclonal antibodies with glycosylation patterns similar or identical to those found in human cells, in the future more monoclonal antibodies are likely to be synthesized in plants rather than in CHO cells. This should allow monoclonal antibodies to be produced in larger amounts and much less expensively than is currently the case.

## 4. Pharmaceutical Production

Given the fact that many human therapeutic agents are commercially produced in animal cells in culture, the resultant proteins end up being relatively expensive. This is because animal cells in culture grow slowly, do not produce a large biomass, and require specialized fermentation media and equipment. As an alternative to animal cells, scientists have begun to produce a number of therapeutic proteins in genetically engineered plants (in a process that is euphemistically called pharming). A few of these plant-synthesized pharmaceuticals have been approved for human use while several others are currently being tested in clinical trials (Table 2). For example, one group of scientists showed that transgenic rice could simultaneously produce three functional proteins which together could neutralize HIV-1 [57]. These proteins included the monoclonal antibody 2G12, and the lectins griffithsin and cyanovirin-N. This protein mixture enhanced both the activity and the binding of the antibody to the HIV protein gp120, resulting in the virus’s neutralization. Thus, this procedure not only decreased the cost of producing this anti-HIV cocktail, but it also increased the potency of the individual components.

Within the next 15–20 years, many more pharmaceutical proteins are likely to be produced in transgenic plants. Tobacco plants are presently the most popular host for the synthesis of pharmaceutical proteins because of (i) the ease of genetically transforming these plants, (ii) tobacco’s rapid growth, and (ii) the large size of the tobacco leaf, which yields large amounts of the target protein [70]. However, in an effort to determine the optimum plant host for foreign proteins, scientists are currently expressing pharmaceutical proteins in a range of different plants. Other plants that have been used to produce foreign proteins include maize, wheat, tomato, potato, mustard, banana, rice, and soybean. For example, some pharmaceutical proteins have been synthesized in rice kernels, which are subsequently ground into a powder and put into easy-to-deliver and consume gelatin capsules. It is important to keep in mind that the purification of plant-produced proteins may present unique challenges and is certainly expected to be different from purifying the same protein from animal cells in culture.

Using traditional techniques (i.e., *Agrobacterium tumefaciens’* transformation of plants) to produce pharmaceutical proteins may be effective; however, it is a relatively time-consuming and labor-intensive process [71]. On the other hand, this approach is currently being replaced by transient expression systems as well as the use of plant cell cultures [71] wherein the time to produce a foreign protein may be reduced by 10-fold or more. Unfortunately, these approaches are currently significantly more expensive than relying on *A. tumefaciens’* transformation followed by plant growth.

As a consequence of concerns among both the public and the scientific community that transgenic plants producing pharmaceuticals could threaten traditional agriculture, the environment and/or human health, it is currently necessary to grow these plants under contained conditions [72]. However, using transient expression systems as well as plant cell cultures should enable scientists to safely produce large amounts of pharmaceuticals in plants.

## 5. Vaccine Production

Antigens that are the basis for conventional vaccines may be produced (similar to various protein pharmaceuticals) in transgenic plants under contained conditions or using either transient expression systems or plant cell cultures. In many countries, conventional vaccines are often too expensive to allow for widespread dissemination, even with the decreased cost of producing the vaccine antigen(s) in transgenic plants. With COVID-19 vaccines, despite donations from wealthy nations, by mid-2021, fewer than “1% of people in low-income countries are fully vaccinated, and just 10% are in lower-middle-income countries, compared with more than half in high-income countries” [73]. For COVID-19, as well as many other diseases, it would therefore be advantageous if vaccines could be delivered directly and inexpensively to individuals in countries that do not have a sufficient infrastructure of modern roads and refrigeration. One way to do this is to produce vaccines in plants that are edible (by humans or animals) and do not require specialized production facilities, sterilization, packaging, or refrigeration. Moreover, unlike conventional vaccines, edible vaccines do not have to be purified prior to their use.

Most current vaccines are delivered by injection. However, since the majority of pathogens enter the body through mucosal sites [74,75], using mucosal vaccination (including through the use of oral vaccines) instead of vaccination by injection, it is possible to create increased levels of protective immunity. Unfortunately, only a small number of mucosal vaccines have been approved for human use; this is a consequence of the lack of effective mucosal delivery systems. On the other hand, with the rapid development of more plant-based edible vaccines, the dearth of mucosal vaccines will soon be a thing of the past. Following the ingestion of an edible vaccine and its subsequent transit into the small intestine, the vaccine antigen is taken up by M cells that line the intestine. Then the vaccine antigen is transported to other cells in the mucosal immune system including macrophages and B cells. The macrophages bind the vaccine antigen and then display it on the macrophage cell surface to helper T cells. The now “activated” helper T cells secrete small molecules that in turn activate B cells that have bound vaccine antigens to surface-localized antibodies. Once the B cells are activated, they synthesize and release antigen-directed antibodies that can eventually neutralize the antigen [76].

Many of the earliest edible vaccines were produced in potatoes, a plant that is relatively easy to genetically manipulate. However, cooking potatoes to make them more palatable inactivates many of the protein antigens that comprise the vaccine. Researchers have therefore tested a number of other plants as delivery vehicles for edible vaccines. These include bananas, tomatoes, lettuce, carrots, peanuts, corn, and rice (Table 3) [77,78,79,80,81,82,83,84]. It is expected that, within the next few years, some of the edible vaccines that have been successfully tested in small animals will subsequently be tested in humans. Thus, the era of large-scale commercialization of edible vaccines will likely become a reality within the next 10–15 years.

## 6. Increasing Nutritional Content

Humans depend on food not only as a source of energy but also for essential nutrients and minerals that maintain the immune system and a healthy lifestyle. However, it is estimated that micronutrient and mineral deficiencies affect almost 800 million people globally, and the majority are people residing in developing countries [103]. In addition to this, poverty and limited access to nutritional food can lead to several disorders, especially among the female population [104].

The availability of a diverse genetic pool for nutritional improvement has enabled the use of genetic modification techniques to develop crop varieties with increased amounts of essential vitamins and minerals. Of particular note is the development of ‘golden’ crops targeted to improve the diets of populations in developing countries that mostly rely on cereals and starchy roots and tubers. For example, ‘golden’ rice, ‘golden’ potato, and ‘golden’ banana have been engineered to accumulate high levels of provitamin A. In golden rice (the first one of these crops to be developed), manipulations in the endosperm-specific carotenoid pathways led to enhanced production of β-carotene, a precursor to vitamin A in rice. By introduction of genes from two different sources, the phytoene desaturase gene (*ctrl*) from the bacterium *Erwinia uredovora*, the phytoene synthase (*psy*) gene and lycopene β-cyclase (*lyc*) gene from daffodil (*Narcissus pseudonarcissus)*, the β-carotene accumulation in rice endosperm was improved following *Agrobacterium*-mediated transformation [105]. Subsequently, by replacing the daffodil gene with a maize gene (under the control of the glutelin promoter) scientists created a new version of golden rice, i.e., golden rice2 (GR2) that produced up to 23 times more beta carotene compared to the original golden rice, with a total carotenoid level of up to 37 mg/g dry weight in the endosperm, of which 31 mg/g dry weight was β-carotene [106]. In a separate study by Diretto et al. [107], a combination of various approaches boosted the potato carotenoid content. The highest carotenoid and beta-carotene content was reported in the Desiree cultivar by using a ‘mini pathway’ of bacterial origin for carotenoid biosynthesis, i.e., by transforming the Desiree cultivar with three genes encoding phytoene synthase (*CrtB*), phytoene desaturase (*CrtI),* and lycopene beta-cyclase (*lyc*) from *Erwinia* under a tuber-specific promoter. The resulting ‘golden’ tubers had 20-fold more carotenoids (114 mg/g dry weight) and >3000-fold more β-carotene (47 mg/g dry weight) compared to the wild-type plant. According to a recent study, “a 150 g serving of boiled golden potatoes has the potential to contribute 42 and 23% of the daily requirement of retinol activity equivalents (RAE), as well as 34 and 17% of the daily vitamin E requirement for children and women of reproductive age, respectively” [108]. A transgenic Cavendish desert banana, displaying a golden-orange phenotype, was developed in Australia and showed an increase in pro-vitamin A when *ZmPsy1* and *MtPsy2a* phytoene synthase genes were overexpressed in fruit pulp. Subsequent field trials in Australia and Uganda showed that the transgene was stable through three successive generations [109]. Clinical trials on some of the ‘golden crops’ are supportive and can prove to be useful in research programs to address malnutrition [110].

There has been considerable progress in elucidating biosynthetic pathways and identifying human-health-related metabolites and facilitating stacking of human health traits. For instance, maize that was genetically engineered by simultaneously targeting three different biosynthesis pathways contained 169-fold more β-carotene, 6-fold more ascorbate, and double the amount of folate compared to the wild-type controls [111]. In another study, a transgenic rice line expressing *AtNAS1, PvFerritin, CRT1,* and *ZmPSY* genes showed enhanced iron, zinc, and b-carotene levels in the rice endosperm [112]. Such studies have opened the door to introduce multi-nutrient traits in key crops such as rice, banana, cassava, and sorghum [113]. In China, the goal of this research is to use gene technologies to produce rice with enhanced vitamin E, iron, zinc, and folate combined with the golden rice trait, as well as improved grain protein content [114].

Upregulating a biosynthetic pathway by modifying transcription factor (TF) expression is another effective strategy to increase amounts of desirable vitamins. For instance, overexpression of *NAC* TFs that are specific to plants and are involved in many developmental processes have been linked to increases in micronutrients such as zinc and iron. In wheat, NAC-like TF *NAM-B1,* which might be linked to early onset of senescence, increased the concentration of zinc in grains [115]. Desired traits can also be obtained by the use of engineered TFs to control the expression of a target gene, and thus zinc finger TFs were designed to increase the amount of tocopherol by modifying the expression of the endogenous γ-tocopherol methyl transferase gene, resulting in a 20-fold increase in alpha-tocopherol in transgenic *Arabidopsis* compared to the control seeds [116].

There has recently been considerable progress on genetic modification of plants for improved protein production in crops. Genome editing technologies aimed at modifying the amino acid quality and quantity have been developed and sold to consumers. The best known examples of this are the Calyno^TM^ (Calyxt, USA), a high-oleic soybean oil, Nutriterra^TM^ (Nuseed, USA) a canola product with high levels of docosahexaenoic acid (DHA) and Sicilian Rouge^TM^ (Sanatech Seed, Tokyo, Japan) a GABA (γ-aminobutyric acid)-enriched tomato variety [117,118,119].

Some notable achievements in altering carbohydrates that impact gut health by modifying the amylose content of starch include metabolically genetically engineered potato (75% more amylose), and wheat with >70% amylose [120,121]. More recently, high-amylose rice plants have been produced through CRISPR/CaS9 targeted mutagenesis of *SBE11b* and *SB1* genes. Furthermore, knocking out the *SBE11b* gene led to longer amylopectin chains, subsequently improving nutritional properties of starch in the rice grain [122]. CRISPR/CaS 9 gene editing has also been used to improve the digestibility of RFOs (raffinose family oligosaccharides) in soybean. By targeting the *GmGOLS1A* and *GmGOLS1B* genes, researchers were able to realize a 33% decrease in the total RFO content in soybean seeds. Interestingly, in this case there were increases in protein and fat content in some of the mutant lines. Furthermore, the authors observed no differences in the agronomic traits between the mutant and wild-type plants [123].

Recently, anthocyanins have attracted interest due to their potential role in preventing obesity and diabetes [124]. Initially, the mode of action of anthocyanins was attributed directly to their antioxidant properties; however, new reports are now emerging that address their cell-molecular interactions, including modulation of gene expression, signaling, and DNA methylation [125]. Many of the genes involved in anthocyanin biosynthesis are available and this has enabled scientists to enhance plant anthocyanin content. For instance, a high anthocyanin content was achieved in the purple tomato variety by engineering the *ROS1* and *Del* regulatory genes [126]. However, incorporating this trait in rice has been unsuccessful despite being tried by numerous researchers, thus illustrating challenges associated with engineering complex biosynthetic pathways in plant [127]. Nevertheless, one group developed purple endosperm rice by using a multi-gene assembly system called TGSII (TransGene Stacking II) for precise effects in rice using CRISPR/Cas-9 genome editing [128]. This experiment suggests extensive gene modification may be required to confer anthocyanin biosynthesis in target species, and that the difficulties in assembling multiple genes into single transformation vectors may require development of a more effective vector system.

## 7. Modifying Fruits and Flowers

Fruits contribute a large portion of vitamins, minerals, antioxidants, phytochemicals, and fiber to our diet and are integral to a healthy lifestyle. Although fruits are mainly grown for consumption, improving aesthetic qualities such as flavor and color could have a significant impact on nutrition if it shifted eating preferences toward more healthy food choices. In the past few decades, consumers have experienced a significant decline in fruit quality, with flavor being one of their major complaints [129]. Developing flavor and taste improvement traits are therefore important quality and health attributes from a consumer’s perspective; however, growers have put most of their efforts into breeding varieties for high yield, disease resistance, size, postharvest shelf life, and large-scale production [130].

Sugar, organic acids, and volatile chemicals have played a large role in shaping fruit flavor. For example, around 200 volatile chemicals have been identified from various cultivars of musk melon (*Cucumus melo*) that contribute to flavor [131]. Genetic manipulation of flavor-inducing genes has allowed researchers to generate various desired mutations enabling development of fruit varieties with superior flavor. For example, modifying the geraniol synthase gene in tomato (increased terpenoids) and *FaFAD1* gene in strawberry (increased γ-decalactone) subsequently produced tastier fruits [132,133]. More recently, gene silencing has been applied to improve taste and fruit flavor. For example, this approach was shown to be effective in strawberry, where the suppression of ADP-glucose phosphorylase and aldose-6-phosphate reductase genes, respectively, by RNAi increased the sugar content in the flesh of the fruit [134].

Improving quality traits to make some fruit more appealing, with a longer shelf life, has been a key objective of both governments and the private sector. The Flavr Savr^TM^ tomato developed by Calgene (California, USA) was the first genetically engineered fruit to be approved for cultivation and human consumption and it helped pave the way for the development of other genetically modified crops that are now widely available [135]. Pink-flesh pineapples (Pink Glow^TM^DelMonte (California, USA)) were produced by expressing a tangerine phytoene synthase (Psy) gene and suppressing endogenous lycopene β-cyclase (β-Lyc) and lycopene ε-cyclase (ε-Lyc) gene expression using RNAi [136]. Additionally, it was possible to alter endogenous ethylene biosynthesis by suppressing meristem-specific ACC synthase gene expression by using RNAi to reduce precocious flowering in Pink Glow^TM^ [136]. Similarly, by using RNAi to mediate the silencing of the β-carotene hydroxylase gene, it was possible to produce a sweet orange variety that was deep yellow in color. Moreover, the β-carotene content in the pulp of transgenic fruits increased 26-fold, thereby significantly improving the health benefits of the fruit [137] Researchers have also manipulated genes involved in the regulation of the anthocyanin pathway that can influence fruit coloration. This has been described in several species including grape berries, strawberries, and apple, where MYB regulatory genes were shown to undergo anthocyanin synthesis to increase anthocyanin production, allowing further enhancement of fruit colors [138,139,140]. As more mechanisms are emerging, increasing anthocyanin pigmentation to further improve fruit color traits seems plausible.

Postharvest losses are a continuing threat in the production chain and result in decreasing returns and lower profits [141]. The main determinant for the postharvest damage of fruits is the rate of softening, which limits fruit shelf life and increases wastage. Therefore, engineering fruits for traits with better resistance to postharvest losses offers potential value. Increased shelf life can be achieved by engineering resistance to ethylene or by the inhibition of ethylene biosynthesis genes. By silencing the key enzymes of ethylene biosynthesis in apple, a significant improvement in fruit shelf life was observed. Notably, the ethylene-suppressed fruits were firmer than the wild-type controls, suggesting the additional role of ethylene in fruit development [142]. Similarly in pear, kiwifruit, and papaya, mutation of ethylene biosynthesis genes resulted in non-ripening phenotypes [143,144,145]. Other traits that have been successfully modified to improve apples include reducing browning by inactivating polyphenol oxidases (PPOs) and other fruit quality traits. For example, the Arctic^TM^ Apple(Okanagan Specialty Fruits, British Columbia, Canada), was developed by silencing the PPOs and currently there are three commercial varieties available: Arctic^TM^ Golden Delicious, Arctic^TM^ Fuji, and Arctic^TM^ Granny Smith [146,147]. In tomato, traditional breeding for traits to slow ripening has detrimental effects on tomato fruit flavor, color, firmness, and nutrition. However, when scientists used an antisense approach targeting the pectate lyase gene to control ripening in tomato, the resultant transgenic tomato lines showed no difference in fruit firmness or color compared to the control non-transgenic lines. Additionally, the genetically engineered lines also had the same yield of fruit as the control lines, which may also benefit the farmer [148]. In another study, repression of MADS box gene expression (*MaMADS1 MaMADS2*) in banana plants extended plant shelf life, and delayed color development and softening [141]. Thus, by using genetic solutions researchers were able to control ripening in banana, which is an important carbohydrate and nutrient source for millions of people.

The use of newer techniques such as CRISPR/Cas9 genome editing provides innovation in fruit crops as these approaches have the potential in some jurisdictions to circumvent the regulatory constraints generally associated with genetically modified organisms (GMOs) [149]. In fruit crops, researchers have used this technique to introduce precise changes at chosen genomic sites to improve flavor and nutrient content, modified flowering, and ripening times. For example, a tomato plant with benefits for both the consumer and the farmer was generated by genome editing. The CRISPR/Cas9-edited variety had uniform growth and produced larger (both size and number), tasty, and sweeter fruit. Notably, the researchers were able to increase the nutritional value of the tomato by lycopene enhancement (~500-fold) and greater vitamin C content, both linked to increased health benefits [150]. Many fruits and vegetables are relatively low in vitamin C, which has led to efforts to increase the vitamin C content of these foods through genetic engineering. One approach to increasing the vitamin C content of crops involves the use of transgenes that encode enzymes involved in vitamin C biosynthesis. For example, Bulley et al. [151] have shown in tomato, strawberry, and potato that overexpression of the *GDP-L-galactose phosphorylase (GGP or VTC2)* gene from kiwi fruit leads to increased accumulation of vitamin C.

Early flowering generally enables plants to have more flowers and fruits. For instance, accelerated flowering and early fruit yield were achieved in cultivated tomato plants modified by CRISPR/CaS9-mediated *SELFPRUNING5G(SP5G)* gene editing [152]. These tools are available for development of cultivars adapted to specific geographical locations.

Recently, scientists have focused on improvement and enhancement of quality attributes in ornamental plant species. Plant characteristics such as flower color and postharvest life can be addressed through genetic engineering. In early years, white-colored flowers generated by antisense RNA or RNAi targeting of structural genes, e.g., the chalcone synthase (*CHS*) gene, were produced in several plant species such as petunia, tobacco, and the garden plant *Torenia hybrida* [153,154,155]. In 1991, the Calgene Pacific company developed a rose variety called Blue Moon that was genetically modified to synthesize delphenedin, a pigment found in most blue flowers [156]. Today, work is ongoing to produce GM flowers with a broad color range and other features. Simultaneous targeting of two genes for flower color modification from white to yellow was attempted in African violet, a commercially valuable ornamental plant. Overexpression of chalcone 4-O-glucosyltransferase (*4CGT*) and aureusidin synthase (*AS1*) genes in African violet led to a color change of the flower petals. The flowers of the yellow-colored cultivar accumulated aureusidin 6-o-glucoside (AOG), a yellow-pigmented compound. In this case, both genes are required to induce expression of AOG to change the color of African violet flowers from white to yellow [157].

In a more recent study, mutations in the dihydroflavonol-4-reductase-B (*DFR-B*) gene resulted in white flowers in CRISPR/Cas9-edited *Ipomea nil* (Pharbitis) plants [158]. Furthermore, modification of a carotenoid cleavage dioxygenase (*CCD*) gene in *Ipomea nil* using the same approach caused white petals to turn pale yellow [159]. Ornamentals like petunia and torenia can represent model plants for studying floral color modifications produced by genetic manipulation. Transformation of torenia plants with a CRISPR/Cas9 construct targeting flavanone-3-hydroxylase (*F3H*), a flavonoid biosynthetic gene, resulted in pale blue flowers [160]. Genes responsible for other flower traits, such as enhancing flower longevity, have also been edited. For instance, editing the ethylene biosynthesis gene to decrease the production of ethylene in petunia improved flower longevity in the mutants compared to the wild-type counterparts [161]. Additionally, the transmission of the edited gene was also confirmed in the T1 generation with similar results to those of the T0 mutants. Such studies may accelerate the development and commercialization of genome-edited ornamentals.

The delivery of preassembled Cas9 protein-gRNA ribonucleoproteins (RNPs) into plant cells can also result in genome editing without the integration of the transgene. Thus, scientists were able to deliver RNPs across the protoplast membrane and subsequently generated petunias with modified pale purple-pink color from an engineered protoplast [162]. However, the overall efficiency of this transient system was very low (only 1 out of 67 transformed lines exhibited the desired color). The low efficiency of this system notwithstanding, these studies offer a new mode of gene editing; however, it remains to be seen whether sufficiently high editing efficiencies can be achieved to make this a popular editing process.

## 8. Fungal and Bacterial Resistance

Plant pathogens reduce crop yields by adversely affecting plant growth and development. It has been estimated that diseases globally reduce crop yields by 20–40% [163]. In the past decade, there has been a surge in the use of genome editing technologies in generating crops that are resistant to a wide range of pathogens. Several studies of targeted mutagenesis in crop plants, including deletions, insertions, and replacement of DNA of various sizes at targeted sites, have proven to be a promising approach to improve plant resistance to pathogens.

Developing plant resistance by modifying host S genes such as those belonging to the *mlo* (Mildew Resistant Locus O) gene family has been effective in apple, tomato, barley, and wheat [164]. However, a common disadvantage of S gene mutation is the concomitant negative impact on plant growth and productivity [165,166]. Therefore, a CRISPR/Cas9-induced targeted deletion in the *MLO-B1* locus resulted in a mutant wheat variety (Tamlo-R32) that thrived better and maintained growth and yields while conserving resistance to powdery mildew [167]. In another study conducted by Peng et al. [168] on citrus plants, CRISPR/Cas9-targeted modification of the S gene *CsLOB1* was performed to enhance resistance to citrus canker. Some recent experiments on rice reported the use of CRISPR/Cas9 technology to induce mutagenesis in the promoter region of bacterial blight S genes, *OsSWEET14* and *OsSWEET11* [169]. Antony et al. [170] developed blight-resistant rice plants with an *OsSWEET14* TDNA insertion mutant but when compared to wild-type plants the mutants had smaller seeds. In contrast, resistance against rice blight with no growth defects was detected in the TALE-edited *OsSWEET14* gene in super basmati rice [171]. These results suggest that engineering S genes through genome editing technology is a potential strategy to enhance rice resistance to blight caused by *Xanthomonas oryzae* pv. Oryzae. Thus, with rice production threatened by bacterial blight causing major crop losses, rapid and durable methods are desperately needed.

In addition to the knockout of host genes to improve disease resistance, endophyte-derived genes can serve as additional routes for improvement of wheat traits. In a recent study by Wang et al. [172], the authors identified a Fusarium resistance gene (*Fhb7*)*,* which was shown to be horizontally transferred from an endophytic fungus and conferred resistance to Fusarium head blight (FHB), a significant fungal disease of wheat. The researchers demonstrated that introgression of *Fhb7* into the genome of many commercial wheat cultivars conferred tolerance to FHB without negatively affecting growth yields, suggesting that *Fhb7* is a potential candidate for engineering blight resistance in elite wheat varieties. Similarly, in tomato plants, CRISPR/Cas 9-mediated knockout of the *DMR6* gene enhanced resistance to bacterial pathogens including *P. syringae, P. capsici,* and *Xanthomonas* spp., with no adverse effects on plant development and growth. As more targets are identified, it is expected that there will be many more successful studies on durable and broad-spectrum disease resistance using the CRISPR/Cas9 approach in a wide range of crops.

The effect of overexpression of plant proteins such as ribosome-inactivating proteins (RIPs) on performance of fungal and bacterial pathogens has been investigated [173]. Some interesting examples, such as overexpression of the *PhRIP* gene in transgenic potato, protected the plants against damage from *Botrytis cinerea* and *Rhizoctonia solani,* whereas expressing the RIP *alpha-MMC* gene improved resistance to rice blast fungus in rice [174,175]. Other proteins, including PFLP (plant ferredoxin-like protein) and HRAP (hypersensitive response-assisting proteins), are effective against multiple bacterial pathogens when they are overexpressed in rice, banana, and other species [176,177]. Recently, overexpression of *PFLP* and *HRAP* genes in greenhouse and field-grown bananas indicated that both genes are effective against bacterial wilt caused by *Xanthomonas* spp. [178]. In an earlier study, a combination of both genes did not provide any additional benefits in banana, yet the authors speculate that bananas expressing both genes may be more durable [179]. Furthermore, the disease-resistance trait was passed on to the next generation of transgenic lines. Additionally, the authors noted no difference in the agronomic traits, including yield and flowering of field-grown symptom-free bananas. These examples are helpful in revealing the biological function of plant proteins that can be beneficial when engineering crop tolerance to pathogen attacks.

Harpins may act in extracellular spaces in plant tissue, facilitating recognition by the plant [180]. Harpins are effective against multiple pathogens when overexpressed in tobacco, rice, canola, and cotton [181,182,183,184,185]. The effect of overexpression of harpin_xooc_ encoding the *hrf2* gene on the performance of an oomycete pathogen was investigated by Niu et al. [186]. The results demonstrate that transgenic soybeans expressing the *hrf2* gene showed enhanced resistance to *Phytophthora sojae.* This study provides a valuable insight toward the functional role of the *hrf2* gene in plant defense against *P. sojae,* opening new avenues for understanding other important pathogens as well as subsequently engineering broad-spectrum disease resistance in soybean.

The ability of plants to utilize diverse classes of immune receptors to perceive the presence of pathogenic microbes makes possible the transferring and engineering of these receptors to improve recognition capacities [187]. Recently, Ercoli et al. [188] conducted studies to show how the XA21 receptor in rice recognizes and resists infection caused by *Xanthomonas oryzae*. Other immune receptors have been targeted for modification in potato, apple, and rice. A late blight-resistant potato with a NOD-like receptor (NLR) introduced from a wild relative is currently on the market in the UK [12]. In apples, modification of the *HcrVf2* gene encoding such a receptor conferred resistance against the devastating fungal scab *Venturia inaequalis* [189]. Xu and colleagues [190] showed that rice plants constantly expressing an immune regulator gene, NPR1 (non-expressor of pathogenesis-related genes 1), conferred resistance to bacterial blight but displayed growth defects. However, when the authors controlled NPR1 expression, it resulted in increased accumulation of NPR1 upon pathogen infection, enhancing resistance to bacterial blight without negatively affecting plant growth and grain yield. Although this method enables researchers to obtain plants with strong immunity, durability can be challenging because pathogens are evolving rapidly.

An important strategy in the fight against Verticillium wilt (VW), caused by fungi belonging to the genus *Verticillium*, is to silence genes essential for spore production, hyphal development, and pathogenicity. RNAi-mediated silencing of the *VdRGS*1 gene has been achieved in cotton, resulting in transgenic plants with enhanced resistances to VW. With the help of RNAi technology, Govindrajulu et al. [191] showed that the transgenic lettuce containing a modified construct of highly abundant message #34 (*HAM34*) and cellulose synthase (*CES1*) genes showed resistance against a biotrophic pathogen that causes downy mildew of lettuce. Similarly, a *PsFUZ7* RNAi construct expressed in transgenic wheat conferred strong resistance to wheat stripe rust. Additionally, knocking down the transcription factor gene *OsERF922* (ethylene responsive factor) using RNAi results in increased resistance against the pathogen *Magnoporthe oryzae* [192]. The improvement of rice blast resistance via CRISPR/Cas9, which targeted knockdown of the *OsERF922* gene in a japonica rice variety cultivated in northern China, was reported by Wang et al. [193]. This modification led to no detrimental effects on rice growth and development. Overall, RNAi appears to be a promising approach to control the detrimental effects of many fungi and oomycetes.

## 9. Increasing Plant Yield

One of the main objectives of growing plants, whether the plants are transgenic or non-transgenic, is to increase the yield of the target plant. Moreover, plant yield is important whether the plant is intended to be used as a food or as the source of a cloned protein. In addition, increasing crop yields can decrease the amount of land that is required for agricultural production [194].

In recent years, scientists have developed several unique schemes intended to increase the yield of various plants. For example, one group of scientists developed an approach that they called ‘speed breeding’ that is applicable to essentially all crops [195]. Speed breeding entails extending the plant’s photoperiod, controlling the plant’s growth temperature, and selection of fast-growing seeds. Of course, to more rapidly grow plants, controlling their growth environment requires the extensive use of greenhouses. In this environment, the lighting may be supplemented with artificial electric lamps [196], the photoperiod may be extended [197], and the wavelength of the lighting may be altered [198]. Following these protocols, researchers have reduced the growth cycle of many different plants to an average of half of what it was previously, thereby enabling the controlled growth of additional generations of the same plant [195].

Scientists have developed several different schemes in an effort to increase plant crop yields. For example, traditional varieties of wheat and rice allocate a significant fraction of their resources to producing vegetative tissues rather than grain or reproductive tissues [199]. However, semi-dwarf varieties previously developed by conventional breeding during the so-called green revolution [200] allocate a greater portion of their resources to grain rather than to vegetative (leaf) tissues. By genetic modulation of the levels of the phytohormones gibberellin and brassinosteroid, it should be possible to produce dwarf plant strains that allocate even more resources to grain instead of vegetative tissues, and thereby further increase the grain yield.

In another study, researchers observed that they were able to genetically modify tomato plants to increase their harvest index, which is the ratio of fruit yield to total plant biomass [201]. This was accomplished by introducing a chloroplast-targeted cyanobacterial flavodoxin gene into tomato plants. In this case, the transgenic plants were generally smaller than the wild-type plants with a higher number of tomato fruits per plant. Moreover, the overall yield of tomato fruit could be augmented by increasing the density of plants in the field (i.e., by planting the transgenic plants close together). In a separate study, scientists increased the expression of the maize (corn) MADS-box transcription factor gene *zmm28* by placing this gene under the control of a moderate-level constitutive maize promoter [202]. In field trials, the transgenic plants with increased expression of the *zmm28* gene showed an increase in carbon assimilation, nitrogen utilization, and plant growth, all leading to an increase in grain yield relative to the cultivar of wild-type maize used in this study. Methylation of the N^6^ position of adenosine residues in RNA molecules is common in plants (as well as in other higher eukaryotes), and this modification is believed to regulate RNA processing and metabolism [203]. In a recent experiment, scientists introduced and expressed the human demethylase FTO gene into rice and potato plants [204]. Expression of this transgene resulted in an exceptionally large increase in rice grain yields in plants that were grown in the greenhouse and a smaller, although still highly significant, increase in rice and potato yield and biomass when plants were grown in the field. In this experiment, expression of the transgene caused an increase of root meristem proliferation and tiller bud formation as well as overall plant photosynthetic efficiency and drought tolerance, suggesting that modulating RNA methylation is an effective way to improve plant yield. Scientists who generated transgenic rice by overexpressing the rice gene encoding a plasma membrane H^+^-ATPase 1 gene (i.e., *OSA1*) found that the resultant transgenic plants significantly improved their utilization of nitrogen and carbon resources compared to wild-type [205]. As a result of this manipulation, the transgenic plants showed a large increase in grain yield.

In addition to introducing exogenous genes to increase plant yield, some researchers have used the CRISPR/Cas9 system to modify some of the existing plant genes (i.e., this technique enables scientists to directly alter specific plant genes) to achieve the same ends as genetic transformation [206]. In one instance, in the T2 generation following the genomic modification, three of the four rice plant mutants that were created yielded an increased number of rice grains and had a larger grain size. Also using the CRISPR/Cas9 system, the alteration of multiple genes in a single tomato cultivar worked together to affect the yield of rice. In this case, the CRISPR/Cas9 system was used to edit six independent loci that were important for controlling the yield and productivity in a wild tomato (*Solanum pimpinellifolium*) crop line [150]. When the engineered tomato plant was compared to the wild-type plant, it was observed to have a three-fold increase in fruit size and a ten-fold increase in fruit number. In addition to these examples, researchers have used the CRISPR/Cas9 system to modify an already shortened version of canola (by conventional breeding) so that the plant has more branches, resulting in the formation of more flowers and pods. This was achieved by knocking out genes for receptors that perceive the hormone strigolactone [207]. Another group of researchers used the CRISPR/Cas9 system to knock out the rice *OsPDCD5* gene, which is involved in programmed cell death [208]. Mutating this gene decreased auxin synthesis by the modified plant in addition to lowering gibberellin and cytokinin synthesis and signaling pathways. Moreover, rice that contained mutations in the *OsPDCD5* gene had an increased yield of rice grains.

From the above cursory exploration of some strategies that have been employed to increase plant fruit or grain yield, it is clear that plant yield is controlled by a relatively large number of different genes. The expression of some of these genes may be increased while the expression of others is decreased with the same ultimate result, i.e., the yield is increased. Moreover, in some instances, the addition of foreign genes may also result in an increase in yield. While most of these genetic manipulations have yet to be tested and proven in the field, there is every reason to expect that the approaches described here will eventually lead to plants with much greater fruit, seed, and grain yield than is currently available from wild-type plants, including plants that have been manipulated by traditional breeding techniques.

## 10. Conclusions

Given the current ease and rapidity with which higher plants can be genetically engineered to yield both highly improved plants and plants inexpensively yielding compounds designed to improve human health and life, it is likely that within the next 10–20 years plants will replace most other means of producing these compounds, i.e., the use of bacterial, fungal, and animal cells in culture. In this regard, following extensive testing, the proven safety and efficaciousness of purified compounds will negate the issue of how these compounds are produced. It will, however, become necessary to develop new and different means of growing transgenic plants for the large-scale production of compounds such as pharmaceuticals. It is therefore possible that plants producing specific useful compounds will be grown hydroponically as a means of protecting valuable agricultural land [209].

## Figures and Tables

**Table 1:** Some full-size monoclonal antibodies that have been produced in plants.

Monoclonal Antibody	Target Antigen (or Disease)	References
Rituximab	CD20 (Non-Hodgkin’s Lymphoma)	[49]
Palivizumab	RSV-F (Respiratory Syncytial Virus)	[50]
ZMapp	GP (Ebola)	[51]
2G12	GP120 (Human Immunodeficiency Virus)	[44]
E16	EDIII (West Nile Virus)	[42]
83K7C	PA83 (Anthrax)	[52]
CaroRx (Chimeric IgA/IgG)	Surface antigen I/II of *Streptococcus mutans* (dental caries)	[53]
Engineered version of mouse GD12	“A” subunit of the toxin ricin	[54]
CAP256-VRC (08 and 09)	GP120 (Human Immunodeficiency Virus)	[47]
ICAM1	(Common cold)	[55]
Anti-Ep-CAM	(Cancer)	[56]
CA1 and CB6	Spike protein (SARS-CoV-2)	[48]
SO57	CD64 (Anti-rabies)	[43]

**Table 2:** Some plant-synthesized pharmaceuticals.

Product	Disease	Plant	Clinical Trial Status	Reference
MB66	HIV-1 and HSV-2	Tobacco	Phase I completed	[58]
Zmapp™ (three chimeric monoclonal antibodies)	Ebola virus	Tobacco	Approved by US FDA in October 2020	[51]
VEN BETA	Gastroenteritis	Rice	Preclinical	[59]
VEN120	Inflammatory bowel disease	Rice	Phase II	[59]
Optibumin	Loss of albumin	Rice	Commercialized	[60]
*Vibrio cholerae*	Cholera	Potato	Phase I	[61]
H5N1 influenza virus-like particle	Influenza	*N. benthamiana* (tobacco)	Phase II	[62]
H1N1 influenza virus particle	Influenza	*N. benthamiana* (tobacco)	Phase II	[62]
Cholera toxin B subunit fused to domain II of dengue virus 2	Cholera and dengue virus	Potato	Preclinical	[63]
Taliglucerase alfa	Gaucher disease	Carrot	Approved by US FDA in 2016, commercialized	[64]
*E. coli* Heat-labile toxin B	Diarrhea caused by enterotoxic *E. coli*	Potato	Phase I	[65]
Aprotinin	Reduces surgical bleeding	Corn	Preclinical	[66]
Insulin	Diabetes	Safflower	Preclinical	[67]
Norwalk virus capsid protein	Diarrhea	Potato, tomato	Phase I completed	[68]
ISOkine™	Growth factor	Barley	Commercialized	[69]
Griffithsin, cyanovirin-N	HIV, ebola, SARS-CoV-2	Rice	Preclinical	[57]

Abbreviations: HIV-1, human immunodeficiency virus 1; HSV-2, herpes simplex virus 2.

**Table 3:** Some plant-based oral vaccines and oral booster vaccines.

Disease	Target Antigen	Plant	Reference
Polio	Poliovirus VP1 antigen	Tobacco	[85]
Hemophilia A	Coagulation factor VIII, heavy chain and C2	Tobacco	[86]
Hemophilia A	Factor IX	Tobacco	[87]
Pompe disease	a-Glucosidase	Tobacco	[88]
Hemophilia B	Factor IX	Lettuce	[89]
Viral nervous necrosis (a fish disease)	Nervous necrosis capsid protein	Tobacco	[90]
Enterohemorrhagic *E. coli*	Enterohemorrhagic *E. coli*	Tobacco	[91]
Norwalk virus	Surface protein	Tomato	[92]
Hepatitis B	Hepatitis B surface antigen	Corn, algae, lettuce, tomato, rice, potato	[93]
Avian influenza	Hemagglutinin H5	Arabidopsis	[94]
Anthrax	Anthrax protective agent	Indian mustard	[95]
Rabies	G-protein	Tomato	[96]
Dengue fever	Dengue viral protein	Tobacco	[97]
Measles	M-antigen	Lettuce	[98]
Type-1 diabetes	Glutamic acid decarboxylase	Red bee	[99]
Human papillomavirus	E7 protein	Algae	[100]
Infectious bursitis virus	VP2 protein	Quinoa	[101]
Cholera	*Vibrio cholerae* toxin B subunit	Rice, potato	[102]

## Data Availability

No data were reported in this study.
